# Culturally-informed strategies to address the differential impacts of climate change-related changing environmental conditions on mental health in regional Australia

**DOI:** 10.1016/j.joclim.2026.100702

**Published:** 2026-08-04

**Authors:** Eric Brymer, Royce Willis, Bindi Bennett, Vinathe Sharma-Brymer, Gillian Gould, Amy Lykins, Philip Batterham, Kim Usher, Matthew Leach, Marg Rogers, Helen Clark, Kate McCubbery, Fiona Charlson, Warren Lake

**Affiliations:** a Mental Health and Psychosocial Wellbeing theme, Faculty of Health, Southern Cross University, Australia; b Manna Institute, University of New England, Australia; c Federation University Australia, Australia; d School of Law and Society, University of the Sunshine Coast, Australia; e Faculty of Health, Southern Cross University, Australia; f School of Psychology, University of New England, Australia; g Centre for Mental Health Research, The Australian National University, Canberra, Australia; h Faculty of Medicine & Health, University of New England, NSW, Australia; i Faculty of Health & Community Services, TAFE SA, Adelaide, South Australia, Australia; j School of Education, University of New England, Armidale, Australia; k Queensland Centre for Mental Health Research, University of Queensland, Australia

**Keywords:** Climate change and mental health, Rural and remote regions, Community resilience, Culturally responsive interventions, Social support networks Human-nature relationship, Nature

## Abstract

Australians living in regional, rural, and remote (RRR) communities face unique climate change impacts that exacerbate both financial and mental health challenges. These communities are disproportionately affected due to their dependence on natural resources for economic well-being and their geographical isolation, which limits access to services. Additionally, poorly designed policies and systems often overlook the specific needs of RRR communities, compounding their vulnerability. This paper highlights the importance of developing culturally responsive and context-specific interventions, co-designed with local communities, to mitigate the mental health impacts of climate change. We propose a conceptual framework for sustainable, community-led policy solutions that enhance social and emotional well-being and foster long-term resilience in RRR communities. The focus is on integrating traditional ecological knowledge, cultural sensitivity, and adaptive strategies to address unique vulnerabilities.

## 1 Introduction

The relationship between environmental conditions and mental health has gained increasing attention in recent years, reflecting the deep interconnection between human health and wellbeing, and the natural environment [1]. In Australia, regional (MM2 on the modified Monash Model – ‘within 20km road distance, of a town with a population greater than 50,000’ [2]), rural, and remote (RRR) communities are increasingly and acutely vulnerable to the effects of climate change due to their reliance on natural resources for economic stability, limited access to mental health services, and heightened exposure to extreme weather events such as floods and bushfires [2]. These climate change-driven environmental stressors, compounded by the geographical isolation of these communities, act as risk multipliers, exacerbating pre-existing vulnerabilities, such as socio-economic disadvantage and underdeveloped mental health infrastructure. Despite this escalating risk, current mental health interventions and policies remain critically insufficient to meet the unique needs of RRR communities [2].

As the frequency and intensity of climate change -related events increase, these disruptions form part of what is now called the ‘new normal’ [3] - a reality characterised by ongoing environmental uncertainty and rapid changes in ecological and societal systems. The National Climate Risk Assessment [4] determined that RRR communities will face very high to severe climate risk, impacting fragile supply chains, resources, emergency response systems and personnel, community structures and social cohesion. This new environmental reality is not a temporary crisis but a permanent shift that will require long-term adaptations, both in how individuals cope with these stressors and how societies support them [5]. The psychological toll of this ‘new normal’—including rising levels of eco-anxiety, ecological grief, and post-traumatic stress disorder (PTSD) and other mental health conditions associated with more frequent and severe disasters—urgently demands a transformative overhaul of mental health interventions [6,7]. This paper aims to address this gap by examining the differential mental health impacts of climate change on RRR populations and proposing culturally responsive, context-specific interventions and policies. Through an analysis of existing frameworks and collaborative strategies, the paper offers a path forward for developing sustainable mental health solutions tailored to these vulnerable communities’ needs.

Climate change is the most significant and accelerating threat to public health in the 21^st^ century, and the mental health impacts of climate change are growing rapidly [6].While there is growing recognition of the mental health impacts of climate change, existing research and policy frameworks continue to systematically overlook the acute and escalating challenges faced by RRR communities, instead focusing on whole-of-population or specific groups such as farmers [2]. Gaps in the literature include a lack of long-term data on the mental health outcomes of climate change affected populations, limited exploration of how cultural factors influence resilience, and insufficient integration of Indigenous knowledges into mental health and wellbeing interventions [8].

Furthermore, many existing policies rely on one-size-fits-all approaches that do not account for the socio-economic, geographical, social, and cultural diversity of these communities. Policies also fail to keep pace with the accelerating scale and severity of climate change impacts and the resulting direct and indirect mental health outcomes [9]. This paper seeks to fill these gaps by advocating for targeted, evidence-based solutions co-designed with local communities and culturally tailored to their specific needs. At the same time, this approach also allows us to pose important questions, such as: What theoretical mechanisms underpin the relationship between environmental stressors and mental health? How can culturally informed approaches address the unique needs of vulnerable populations?

In framing this discussion, we acknowledge the United Nations’ definition of mental health as “a state of well-being in which an individual realises his or her own abilities, can cope with the normal stresses of life, can work productively and is able to make a contribution to his or her community” [10]. However, we extend this understanding to recognise that mental health cannot be fully understood without considering the broader environmental and social context—the ‘new normal’ of climate change. This context demands ongoing mental adaptation as individuals face not only direct exposure to environmental disasters but also indirect stress from the continuous media coverage of such events, both traditional and social.

Australia has recently experienced a succession of catastrophic and unprecedented extreme weather events, including droughts, floods, bushfires, and cyclones, which have profoundly impacted communities across the country [11]. These events lead to rising levels of emotional distress, anxiety, and ecological grief, particularly as individuals perceive threats to their environment and way of life [12]. In RRR communities, these experiences are amplified by limited access to support services and increased dependence on the natural world, making urgent culturally grounded, context-specific interventions indispensable for building resilience.

Rural and remote Australia is home to approximately 7 million people (28% of the national population), who experience distinct health and social challenges shaped by geographic isolation and limited service availability [13]. These regions report higher rates of injury, death, preventable hospitalisations, and chronic health risks, including low physical activity, obesity, harmful alcohol use, and higher smoking prevalence. Aboriginal and Torres Strait islanders make up 32% of the population in remote and very remote areas, and cultural diversity varies widely, with 1% to 12% of residents identifying as culturally and linguistically diverse (CALD) or born overseas [[14], [15], [16]]. Compounding these factors, rates of family violence remain disproportionately high for both, women (23% RRR vs 15% urban) and men (6.6% RRR vs 5.9% urban) in RRR communities, and life expectancy trails urban averages by roughly four years [17]. Together, these intersecting health, social and demographic patterns expose entrenched structural inequities that severely compromise everyday wellbeing in RRR communities—conditions that climate change is poised to further strain.

## 2 Risks and impacts of climate change on mental health in RRR settings

Mental health is typically viewed as a continuum ranging from mental ill-health (e.g., depression, anxiety) to optimal mental well-being (e.g., flourishing), with languishing representing the absence of mental illness without the presence of well-being [18]. The interaction between environmental factors and mental health is complex. Research from a variety of fields has found positive mental health outcomes from interacting with natural environment and experiences of being connected to or part of the natural environment [19,20]. While the relationship is complex, recent research has found that higher interactions with nature and stronger feelings of connection to nature can result in higher mental wellbeing even for those impacted by climate change-related mental health issues [21,22] However, shifts in climate, pollution, and climate-driven changes in natural surroundings are exerting significant influence on mental health outcomes [[23], [24], [25], [26], [27]]. Evidence is mounting that climate change, which contributes to increased disasters and extreme weather events, can precipitate conditions such as PTSD, major depressive disorder, anxiety (including eco-anxiety), complicated grief, survivor guilt, substance abuse, suicidal ideation, exacerbating pre-existing mental health issues, and even contributing to suicide risk [7,[27], [28], [29], [30]].

A recent survey revealed that 80% of Australians have been exposed to extreme weather events since 2019, with 51% reporting adverse mental health impacts [31]. Alarmingly, 21% experienced moderate to severe effects on their mental well-being. These impacts are disproportionately felt in RRR communities [31], characterised by their diverse ecosystems and geographic isolation.

The mental health impacts of climate change in RRR communities arise from several interconnected mechanisms. Prolonged exposure to climate change driven environmental stressors, such as recurring floods or droughts, can activate chronic stress responses, leading to mental health conditions like PTSD and anxiety [32]. In these RRR areas, the economic instability caused by the loss of natural resources or agricultural failure further contributes to stress, financial insecurity, and an increased risk of depression or suicidal ideation [30]. Social isolation, whether due to displacement or loss of community ties, compounds the effects by eroding critical support networks [17,33]. While evidence is insufficient to draw definite conclusions [34], for Aboriginal and Torres Strait Islander peoples, whose social and emotional wellbeing is deeply intertwined with belonging to Country [35], the cultural dislocation caused by climate change can lead to profound psychological distress, including experiences akin to solastalgia – the grief and sadness arising from environmental loss and change which is positively associated with depression, anxiety and PTSD [36,37]. Moreover, the rising phenomenon of eco-anxiety, particularly among younger generations, reflects a growing sense of helplessness and fear for the future, leading to chronic stress and emotional exhaustion [38].

### 2.1 Climate change and the intersections of vulnerability

The mental health impacts of climate change in RRR communities cannot be fully understood without considering the intersections of vulnerability—how multiple, overlapping factors compound psychological distress. In RRR communities, socio-economic disadvantage, pre-existing mental health conditions, and geographical isolation often intersect to exacerbate the mental health impacts of climate change-driven environmental stressors [39]. For instance, individuals facing economic insecurity may also lack access to mental health services due to geographic isolation, creating a cycle of cumulative stress that intensifies their psychological burden. Similarly, those with pre-existing mental health conditions may find their symptoms worsened by climate change-related stressors, particularly if they also face housing insecurity or poverty. Indigenous communities, whose deep cultural and spiritual connection to the land is integral to their identity, experience a unique intersection of cultural dislocation and environmental loss, which amplifies the psychological impacts of climate change [36,40]. This disruption threatens their sense of place and exacerbates existing socio-economic inequalities. Gender and caregiving responsibilities further intersect with these vulnerabilities, particularly for women in low-income households, who face additional burdens during climate change-related crises (such as increased unpaid caregiving labour due to disruption to schools, health services and community supports, and a consequential reduction in capacity for paid employment) [[41], [42], [43]]. Older adults face specific health risks from climate change-related extreme heat, flooding, drought, and other climate hazards (such as dehydration, exacerbation of cardiovascular and respiratory conditions, reduced mobility during evacuation or service disruptions, and social isolation) that require targeted strategies to support mitigation, adaptation and anticipation [44,45]. Another high priority subgroup is pregnant women, and especially those who smoke tobacco, who are more susceptible to adverse sequalae from poor air quality, such as from bush fires, with adverse effects such as giving birth to infants with low birth weight [46,47].

Young people, sensitive to societal and environmental changes, frequently report heightened anxiety and stress related to uncertain environmental futures [48]. As they face the prospect of inheriting a world marked by climate instability, they often experience eco-anxiety—a chronic concern about the future of the planet. This anxiety can be compounded by feelings of helplessness and frustration over perceived inaction by older generations, which can intensify their mental health challenges.

Farmers are acutely affected by climate change-driven variability and extreme weather events (such as decreased crop yield, destocking and disposal of livestock and undermined self-identity), often facing both financial and psychological distress [[49], [50], [51]]. Meanwhile, multicultural populations, including migrants and humanitarian entrants, contend with unique challenges, such as acculturation stress and difficulties accessing culturally appropriate mental health resources, which may leave them more vulnerable during climate change driven environmental crises such as extreme heat [52].

First responder and military families, who frequently relocate, may be less embedded in local communities [53] and therefore experienced greater social isolation than longer-term residents [54]. This may hinder their ability to utilise local support networks or protective environmental factors that could mitigate the psychological toll of climate disasters. Without deep community ties or knowledge of local resources, these families may be more susceptible to the mental health impacts of environmental events.

These vulnerabilities underscore the necessity for a deeper conceptual understanding of how interventions mediate the relationship between environmental changes and mental health in a culturally responsive way. Whether it is Aboriginal and Torres Strait Islander communities dealing with disruptions to their traditional ways of life or first responders struggling to integrate into local support networks, mental health solutions may have greater relevance and effectiveness if they are co-designed with communities.

## 3 Addressing the needs of priority populations

Certain groups within RRR Australia are particularly vulnerable to the compounded effects of climate change environmental factors and poorly designed policies. Studies have demonstrated that these populations are often more susceptible to mental health challenges following extreme weather events [55]. These groups include young people, Aboriginal and Torres Strait Islander communities, multicultural populations, women (especially pregnant women), people living with disabilities, low-income individuals, those facing housing insecurity, and people with pre-existing mental health conditions such as anxiety and depression.

Interventions with cultural responsiveness and context-specific approaches are critical when working with Aboriginal and Torres Strait Islander peoples and multicultural and minoritised communities and are applicable to both groups. Indigenous research methodologies such as Aboriginal Participator Action Research (APAR) prioritise the centrality of First Nations people’s involvement in research with their knowledges, respect for culture, and self-determination [56,57]. Collaborative efforts with Aboriginal and Torres Strait Islander communities, for example, can integrate traditional ecological knowledge with contemporary mental health research, fostering both resilience and well-being [58,59]. Interventions developed through these co-design processes include an Indigenous model of mental health care and tailored digital treatment programs [34], which aim to be more culturally relevant, contextually appropriate, and grounded in local knowledge systems. They are tailored to the specific needs of these communities. This raises important conceptual questions: How can these knowledge systems integrate to create unified models of resilience? What theoretical insights can be drawn from their interplay? In addition, implementing culturally responsive interventions in under-resourced RRR communities has practical challenges, including funding constraints, workforce shortages, and the assurance of cultural sensitivity. Overcoming these challenges requires strategic investments in community-driven programs, capacity building, telehealth infrastructure, and a long-term commitment to cultural humility and trust building [59]. By addressing these feasibility concerns, governments and local organisations can ensure that culturally responsive mental health interventions are practical and sustainable, leading to better mental health outcomes for RRR populations. Traditional ecological knowledge also provides valuable insights into land management, environmental stewardship, and community connection to the natural world, which can be leveraged to improve mental health outcomes [60].

Similarly critical is developing culturally sensitive and responsive interventions for Australia’s multicultural populations. These interventions should include language-appropriate resources, mental health supports that reflect diverse cultural values, and an understanding of varied coping mechanisms [61]. For instance, providing mental health services in a range of languages, incorporating spiritual or community healing practices, or using culturally specific metaphors in therapy can improve the accessibility and effectiveness of interventions [62]. Co-design approaches such as storytelling with the creation of storyworlds are collective, community-led, and culturally sensitive [63]. Such transdisciplinary methodologies enhance awareness on local-specific climate adaptation supporting interventions. Other dimensions of diversity may also be important to consider when developing interventions for RRR Australia, including sociodemographic, developmental, workforce, service delivery, and funding factors. Designing interventions should consider community-based participatory approaches [64]. For example, interventions designed for regional youth are more successful when young people are included as stakeholders throughout the development and implementation stages. Involving youth ensures that programs are relevant to their unique experiences and challenges, as well as being feasible and likely to be widely accepted. Engaging young people in co-design processes can clarify resources already in place and lead to more innovative and targeted support mechanisms [65], enhancing mental health literacy and resilience in the face of climate change driven environmental challenges.

Considering multiple dimensions of cultural, environmental, developmental and sociodemographic needs is important for adapting or bolstering existing services creating services and interventions that are acceptable, effective, engaging and sustainable. Working with local communities to account for local needs is crucial for ensuring that solutions are fit for purpose, making them more likely to resonate with and benefit participants.

## 4 Towards a framework for addressing effects of climate change on RRR mental health

**Fig. 1:**
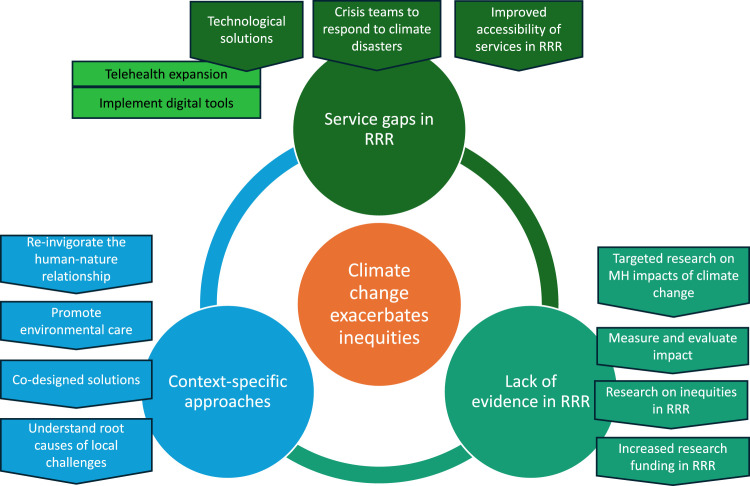
Working framework to address climate change impacts on mental health in RRR communities.

Systemic changes necessary for sustainable mental health interventions in RRR communities include long term strategies as well an urgent need for immediate, short-term solutions to address current mental health challenges (see below). Informed by knowledge-to-action frameworks [66], we propose a working framework (Fig. 1) for addressing the mental health effects of climate change. Developing the necessary evidence base to assess and modify climate change effects on mental health requires a clear understanding of the gaps in RRR services, and knowledge of the best locally tailored approaches for addressing climate change driven environmental change. The availability of targeted evidence on the challenges faced by RRR communities can guide more effective action to mitigate climate change driven environmental change and create bespoke supports that meet the needs of RRR communities. Such action needs to layer individual, interpersonal, community and societal/political change [67] to ensure that solutions can sustainably address the complex drivers of both mental health and the rural environment. Further work is needed to operationalise and test the framework in action; however, a working framework may be useful for guiding research activity, community action, policy and practice around key potential solutions that may improve mental health outcomes in RRR communities. Below, we provide further detail on potential solutions proposed within the framework.

## 5 Mental health service solutions

Working to improve accessibility and quality of mental health services involves short- and longer-term solutions. Short-term solutions serve as useful components as part of a longer-term strategy within a broader service system that addresses individual, service, community-level and system-level barriers to accessing services, including mental health literacy, risk communication, stigma, and capacity building of MH professionals. This ensures that populations that experience vulnerability receive timely support in the face of climate change related mental health challenges. Implementing the actions now can mitigate immediate distress while setting the stage for more sustainable mental health solutions in the future. They also serve as critical opportunities for gathering data to inform the iterative development of long-term frameworks.

Short term service solutions required to ensure sustainability include:

a) **Expanding telehealth services**: Scaling up telehealth infrastructure to provide immediate access to mental health professionals in remote areas [68]**.**
b) **Deploying mental health crisis teams**: Mobilising mental health professionals to provide on-the-ground support in communities experiencing extreme weather events might be useful. However, evidence also suggests that some interventions might exacerbate mental distress [69].
c) **Delivering evidence-based digital tools**: Delivering online resources and mobile apps [70] (culturally adapted or co-designed) that have been shown to be effective in providing coping strategies and mental health support to communities affected by climate events.

Long term service solutions required to ensure sustainability include:

a) **Improve Access to Mental Health Services:** RRR communities experience significant barriers to accessing mental health care. We recommend sustainably expanding services (e.g., psychosocial and preventive services that make the best use of local natural environments, telehealth or mobile health clinics, or community and virtual support groups) to ensure equitable access to care. Strengthening social and relational capital within communities can also help build resilience to the mental health impacts of climate change.
b) **Move Beyond Resilience and Adaptation:** While resilience and adaptation are essential, they may not be sufficient for RRR communities facing compounding disadvantages. We propose community-driven and implemented prevention and promotion programs that focus on the root causes of localised community problems, e.g., ecosystem restoration, rather than just supporting communities to address mental distress. These programs should be co-designed with RRR populations and subgroups, ensuring they are locally relevant and sustainable.
c) **Reinvigorate the Human-Nature Relationship:** Strengthening the human-nature connection is key to improving mental and physical health outcomes. Programs encouraging engagement with nature, such as land-care initiatives and nature-based interventions, should be encouraged and evaluated. These should also promote the integration of Indigenous knowledge, such as “Caring for Country,” to enhance the human-nature relationship in ways that are meaningful to local communities.
d) **Promote Environmental Care as a Core Strategy:** Mental health solutions must recognise the interdependence of humans and the environment. Research and intervention programs should advocate for environmental care as a central component of mental health strategies, focusing on behaviours that promote environmental sustainability and community well-being.

## 6 Research and data solutions

To ensure the short- and long-term mental health interventions are evidence-based and appropriate across RRR communities, the effective design and implementation of research is essential. This may require more careful attention to traditional ecological models as well as co-design principles. We propose the following important concepts:

a) **Research needs to be targeted:** Current evidence highlights the mental health impacts of climate change on RRR communities, particularly through direct experiences with extreme weather events and indirect stressors like ecological anxiety. However, long-term, high-quality data are lacking. We recommend establishing a national longitudinal study to track and respond to mental health outcomes in these populations over time, particularly as climate change accelerates.
b) **Research needs to address inequities in impact:** Climate change disproportionately affects disadvantaged groups, including those in RRR areas, across all age groups. Research should prioritise rigorous evaluation of interventions that address the specific vulnerabilities of these communities, with a focus on tailored mental health programs designed to address the unique cultural, social, and economic challenges they face. Implementation research is also needed to inform optimal strategies for disseminating evidence-based interventions into RRR communities.
c) **Design Fit-for-Purpose Research and Integrated Interventions:** Effective research and interventions for RRR communities must consider community demographic, social, and psychological factors unique to these populations. Co-designing with local communities can foster culturally appropriate and context-specific interventions, integrating Indigenous knowledge and land care practices where relevant. Consideration needs to be given to Indigenous data sovereignty.
d) **Measure and Evaluate Impact:** Rigorous, evidence-based evaluations of mental health interventions are essential. Research must utilise validated outcomes, adopt suitable and rigorous designs (e.g., randomised control trials, longitudinal studies) as well as those valuable for Indigenous peoples through partnership at all stages of the data lifecycle [71,72], and participatory methods to ensure interventions are effective and replicable across RRR contexts.

## 7 Solutions for strengthening communities and addressing policy gaps

The interventions discussed, including the integration of traditional ecological knowledge and the co-design of mental health solutions, highlight the importance of policies that prioritise culturally responsive, community-led and context-specific approaches. Recent national policy developments, including the National Health and Climate Strategy and the National Climate Risk Assessment, provide an important foundation for action. However, gaps within these frameworks limit their capacity to effectively respond to the mental health impacts of climate change in RRR communities and therefore require targeted policy attention.

While the National Health and Climate Strategy represents a significant step forward in recognising climate-related health risks, including mental health, it remains largely oriented towards national coordination and system-level reform. A key limitation is the absence of clearly articulated mechanisms for sustained local community engagement, particularly in RRR contexts. Without explicit support for community co-design, local governance capacity and place-based implementation, there is a risk that policy responses remain high-level and insufficiently responsive to the social, cultural and environmental realities of RRR communities.

Similarly, the National Climate Risk Assessment identifies RRR communities as facing very high to severe climate risk, including risks to health and social systems. However, its primary focus on biophysical hazards and system-level impacts affords limited attention to psychosocial and mental health consequences, such as cumulative trauma, ecological grief and eco-anxiety arising from repeated climate events. In addition, the national and regional scale of analysis may obscure local variation, reducing the usefulness of the Assessment for place-based mental health planning in diverse RRR settings.

To address the specific needs of these populations (such as young people, Aboriginal and Torres Strait Islander peoples, first responders, defence personnel, multicultural and LGBTIQA+ communities), and to ensure that national policy frameworks translate into meaningful local outcomes, we propose the following actionable policy recommendations:

a) **Adopt Context-Specific Approaches:** While traditional mental health interventions can be effective, one-size-fits-all approaches are unlikely to meet the needs of RRR populations. Policies should explicitly support evidence-based, locally tailored interventions that are co-designed with communities and responsive to cultural, environmental and service-context diversity.
b) **Support Increased and Interdisciplinary Funding:** Governments and funding bodies should develop policies that prioritise sustained, interdisciplinary, cross-institutional investment in research and intervention design that strengthens local service capacity, supports community-led initiatives, and enables longitudinal data collection in RRR areas. This includes funding for environmental and health monitoring infrastructure to support locally relevant planning, evaluation and adaptation over time.

## 8 Conclusion

A commitment to advancing the discourse on community vulnerability and resilience is paramount in unravelling the intricate relationship between, and responding to the effects of, changing environmental conditions and mental health in RRR Australia. This paper highlights the importance of conceptualising mental health and wellbeing not just as an outcome but as a dynamic process that is informed by cultural, environmental, and psychological dimensions. We propose a working framework to support ongoing action for better understanding and responding to this process.

As climate change accelerates, bringing with it increasing heat and frequency and severity of extreme weather events, so too does the urgency to address the challenges of building mental health capacity and resilience in vulnerable regions. However, as climate change has been recognised as the greatest public health concern across the globe for over a decade, this urgency has now become critical. By acknowledging the unique vulnerabilities faced by groups disproportionately affected by climate change, such as Indigenous and CALD populations, this paper underscores how to respond to these pressing needs through culturally informed, context-specific approaches, paving the way for interventions and policies that are responsive to cultural contexts, conducive to mental health and well-being, and sensitive to the human-nature relationship.

Given the ‘new normal’ of environmental uncertainty, mental health frameworks must evolve to address the ongoing impacts of climate change on RRR communities. Integrating traditional ecological knowledge, community-driven solutions, and context-specific approaches may be critical for building resilience and ensuring that mental health interventions are sustainable and effective. Policymakers must act now to implement these strategies and ensure that the most vulnerable populations are not left behind in the climate response. Ultimately, a more inclusive and comprehensive approach to mental health in the dynamic landscape of rural and regional Australia will require creating adaptable, culturally relevant, and scalable frameworks and solutions across diverse communities.

## CRediT authorship contribution statement

**Eric Brymer:** Writing – review & editing, Writing – original draft, Conceptualization. **Royce Willis:** Writing – review & editing, Writing – original draft, Conceptualization. **Bindi Bennett:** Writing – review & editing, Writing – original draft, Conceptualization. **Vinathe Sharma-Brymer:** Writing – review & editing, Writing – original draft, Conceptualization. **Gillian Gould:** Writing – review & editing, Writing – original draft, Conceptualization. **Amy Lykins:** Writing – review & editing, Writing – original draft, Conceptualization. **Philip Batterham:** Writing – review & editing, Writing – original draft, Conceptualization. **Kim Usher:** Writing – review & editing, Writing – original draft, Conceptualization. **Matthew Leach:** Writing – review & editing, Writing – original draft, Conceptualization. **Marg Rogers:** Writing – review & editing, Writing – original draft, Conceptualization. **Helen Clark:** Writing – review & editing, Writing – original draft, Conceptualization. **Kate McCubbery:** Writing – review & editing, Writing – original draft, Conceptualization. **Fiona Charlson:** Writing – review & editing, Writing – original draft, Conceptualization. **Warren Lake:** Writing – review & editing, Writing – original draft, Conceptualization.

## Declaration of competing interest

There are no conflicts of interest
